# Assessment of non-invasive ICP during CSF infusion test: an approach with transcranial Doppler

**DOI:** 10.1007/s00701-015-2661-8

**Published:** 2015-12-23

**Authors:** D. Cardim, M. Czosnyka, J. Donnelly, C. Robba, B. C. T. Cabella, X. Liu, M. T. Cabeleira, P. Smielewsky, C. Haubrich, M. R. Garnett, J. D. Pickard, Z. Czosnyka

**Affiliations:** Brain Physics Laboratory, Division of Neurosurgery, Department of Clinical Neurosciences, University of Cambridge, Box 167, Cambridge Biomedical Campus, Cambridge, CB2 0QQ UK; Neurosciences Critical Care Unit, Addenbrooke’s Hospital, Cambridge University Hospitals NHS Foundation Trust, Cambridge, UK; Department of Neurology, University Hospital Aachen, Aachen, Germany; Division of Neurosurgery, Addenbrooke’s Hospital, Cambridge University Hospitals NHS Foundation Trust, Cambridge, UK; Institute of Electronic Systems, Warsaw University of Technology, Warsaw, Poland

**Keywords:** Non-invasive ICP monitoring, Transcranial Doppler, Cerebral blood flow velocity, CSF infusion test

## Abstract

**Background:**

This study aimed to compare four non-invasive intracranial pressure (nICP) methods in a prospective cohort of hydrocephalus patients whose cerebrospinal fluid dynamics was investigated using infusion tests involving controllable test-rise of ICP.

**Method:**

Cerebral blood flow velocity (FV), ICP and non-invasive arterial blood pressure (ABP) were recorded in 53 patients diagnosed for hydrocephalus. Non-invasive ICP methods were based on: (1) interaction between FV and ABP using black-box model (*nICP_BB*); (2) diastolic FV (*nICP_FVd*); (3) critical closing pressure (*nICP_CrCP*); (4) transcranial Doppler-derived pulsatility index (*nICP_PI*). Correlation between rise in ICP (∆ICP) and ∆nICP and averaged correlations for changes in time between ICP and nICP during infusion test were investigated.

**Results:**

From baseline to plateau, all nICP estimators increased significantly. Correlations between ∆ICP and ∆nICP were better represented by *nICP_PI* and *nICP_BB*: 0.45 and 0.30 (*p* < 0.05). *nICP_FVd* and *nICP_CrCP* presented non-significant correlations: −0.17 (*p* = 0.21), 0.21 (*p* = 0.13). For changes in ICP during individual infusion test *nICP_PI*, *nICP_BB* and *nICP_FVd* presented similar correlations with ICP: 0.39 ± 0.40, 0.39 ± 0.43 and 0.35 ± 0.41 respectively. However, *nICP_CrCP* presented a weaker correlation (*R* = 0.29 ± 0.24).

**Conclusions:**

Out of the four methods, *nICP_PI* was the one with best performance for predicting changes in ∆ICP during infusion test, followed by *nICP_BB*. Unreliable correlations were shown by *nICP_FVd* and *nICP_CrCP*. Changes of ICP observed during the test were expressed by nICP values with only moderate correlations.

## Introduction

Intracranial pressure (ICP) monitoring is indicated in a wide range of neurological diseases. However, apart from major trauma and academic neurosurgical centres, it is not commonly part of the clinical management of patients. This scenario is mainly attributed to the invasiveness of the current methods (which require insertion of a catheter into the brain) and their associated risks (infections, brain parenchyma damage and haemorrhages). Such characteristics limit ICP monitoring in several clinical conditions in which ICP would be important: patients with haemorrhagic or ischaemic stroke, mild to moderate traumatic brain injury (TBI), altered mental status or cognitive/psychological disorders, brain tumours and hydrocephalus [7, 8, 11]. As an effort to provide alternatives for ICP monitoring, some methods have been proposed to measure and monitor ICP non-invasively (nICP).

Transcranial Doppler (TCD), for instance, is primarily a technique for diagnosing various intracranial vascular disorders such as emboli, stenosis or vasospasm [15], but has been broadly utilised for non-invasive ICP monitoring due to its ability to detect changes in cerebral blood flow velocity (FV) derived from ICP variations. TCD has also been used for non-invasive estimation of cerebral perfusion pressure (CPP), based on some parameters derived from FV, such as diastolic flow velocity (FVd) [6] and critical closing pressure (CrCP) [17]. Considering the assumption that CPP equals the difference of arterial blood pressure (ABP) and ICP, nICP can be estimated as **nICP** = **ABP** – **nCPP**.

Infusion test is a diagnostic modality that enables assessment of the cerebrospinal fluid (CSF) dynamics and the compensatory ability of the cranial-spinal fluid system in patients diagnosed with hydrocephalus [18]. The principle of this test is based on a constant infusion rate of artificial CSF into any accessible CSF compartment, which produces a gradual and uncompensated increase in ICP [10]. Our objective in this study was to compare the estimation performances of four TCD-based nICP methods in cohort of hydrocephalus patients whose CSF dynamics were investigated using infusion tests involving controllable test-rise of ICP.

## Materials and methods

### Patients

We retrospectively analysed data collected during the period of 1994–1998 and 2006 at Addenbrooke’s Hospital, Cambridge, United Kingdom. The population consisted of 53 patients who undertook an infusion test in order to investigate CSF disturbances. Measurements included ICP, ABP and FV in middle cerebral artery (MCA). The median age of the patients was 55 years [interquartile range (IQR), 66–38 years; 31 men, 22 women].

### Infusion test

An infusion test is a computerised method involving cerebrospinal infusion of artificial CSF (normal saline or Hartman’s solution) into the subarachnoid space, based on the traditional constant rate infusion test described by Katzman and Hussey [10]. For this purpose, one spinal needle (18 G, 3.50 in.) is inserted between the lumbar L3/L4 or L4/L5 vertebrae, and it is used for both pressure measurements and fluid infusion. This needle is connected to a pressure transducer via a stiff saline-filled tube and to an infusion device (Alaris® GH Plus Syringe Pump; Carefusion, Basingstoke, UK) with a 50-mL syringe (BD, Oxford, UK), mounted on a trolley containing a pressure monitor (Simonsen and Will, Sidcup, England) connected to a computer [5]. The threshold of the opening pressure is 13 mmHg and the infusion rate is 1 mL/min when the pressure is higher than the threshold. Conversely, the infusion rate is 1.5 mL/min when the pressure is lower than the threshold. The start of infusion is initiated after baseline recording (10 min), whereas the end of the infusion is signalled by the achievement of a steady-state ICP plateau. If ICP rises above 40 mmHg, the infusion needs to be discontinued to avoid excessive elevation of the pressure above the safe clinical limits [18]. After cessation of the infusion, ICP is continuously recorded until it decreases to a steady baseline level [5].

### Data acquisition

The ICP measurements were made with the patient placed in a lateral position (left or right), considering the foramen of Monro as the zero calibration reference. TCD ultrasonography (Neuroguard; Medasonics, Fremont, CA, USA) was used for monitoring of blood FV in the MCA, through a 2-MHz probe fixed on the cranium by using a commercially available headband. ABP was recorded non-invasively by using a Finapres® finger cuff (Ohmeda, Englewood, CO, USA) positioned at the level of the heart. Analogue outputs of ICP and ABP from the pressure monitors and the TCD monitor were connected to an analogue-to-digital converter (DT2814; Data Translation, Marlboro, CA, USA) fitted into an IBM-compatible laptop computer (Amstrad ALT 386 SX; Amstrad, Brentwood, UK). Data were sampled (50 Hz), digitised and stored on the hard disk using software for waveform recording (WREC; W. Zabolotny, Warsaw University of Technology, Warsaw, Poland). The recorded signals were then analysed using ICM+ software (Cambridge Enterprise, http://www.neurosurg.cam.ac.uk/icmplus). Informed consent was obtained from all individual participants included in the study (or their next of kin) for the use of collected data for research purposes. The infusion test is a routine clinical investigation in the Hydrocephalus Clinic, Addenbrooke’s Hospital, Cambridge, with no ethical approval required. The data were analysed anonymously as a part of a clinical audit.

### Non-invasive ICP methods

The four methods used for nICP estimation in this study were:

1. Schmidt et al. [14] “black-box” (BB) model (*nICP_BB*): nICP is obtained from a mathematical “black-box” model based on the presumed transformation between ABP and ICP waveforms. Coefficients of these transformations are derived from the database of real ABP and ICP recordings. Similar linear transformation is built, using the same database between FV and ABP. Then, the model assumes a linear relationship between ABP and FV and ABP to ICP transformations. Multiple regression coefficients are calculated. Finally, for each prospective study, ICP is calculated using ABP to ICP transformation, formed from ABP to FV transformation transposed using precalculated regression coefficients. Non-invasive ICP estimation using this method was performed using a plugin developed for ICM+ software.
2. Czosnyka et al. [6] (*nICP_FVd*): for this method, based on the diastolic flow velocity for the estimation of nCPP, nICP was calculated as the difference between ABP and nCPP (nICP = ABP - nCPP). The equation for nCPP estimation is:

1 $$ nCPP=ABP\times \frac{FVd}{FVm}+14 $$

*FVm* and *FVd* represent mean and diastolic flow velocity, respectively.

3. Varsos et al. [17] (*nICP_CrCP*): this method also calculates nICP based on nCPP, in this case specifically using the concept of critical closing pressure (CrCP). The equation for nCPP estimation is:

2 $$ nCPP=ABP\times \left[0.734-\frac{0.266}{\sqrt{{\left(CVR\times Ca\times HR\times 2\pi \right)}^2+1}}\right]-7.026 $$

*CVR* represents cerebral vascular resistance, *Ca* denotes arterial compliance of the cerebral bed and *HR* expresses heart rate (beats/s), with ABP and FV as the required measurements. Constant coefficients (0.734, 0.266, 7.026) are derived from analysis of database of 232 TBI retrospective cases [17].

4. *nICP_PI*: nICP estimation based on TCD-derived pulsatility index was based on the linear regression among known values of ICP and pulsatility index (PI) from a population cohort of 292 TBI patients. The regression equation was based on data analysed by Budohoski et al. [2] and given by:

3 $$ nICP=4.47\times PI+12.68 $$

4 $$ PI=\frac{F{V}_s-F{V}_d}{F{V}_m} $$

*FVs*, *FVd* and *FVm* represent systolic, diastolic and mean flow velocity.

### Statistical analysis

Statistical analysis of the data was conducted with OriginPro statistical software (version 8; OriginLab, Northampton, MA, USA). Data were tested for normal distribution using Shapiro-Wilk test. The analysis included Spearman correlations between mean ∆ICP and ∆nICP and averaged correlations for variations of nICP across time during ICP increase. The ∆ value (magnitude) was considered the difference between plateau and baseline mean values in each recording during infusion. *R* represents the Spearman correlation coefficient, with the level of significance set at 0.05. The Bland-Altman method was used to determine the agreement between measured ICP and the different nICP methods, with their respective 95 % CI for prediction of ICP and bias for baseline and plateau periods. The confidence interval represents the range of values around the bias (difference between mean values of nICP and ICP), in which data can be found with a significance level of 0.05. In addition, Mann–Whitney test was used to assess whether nICP samples originate from the same distribution as ICP for both baseline and plateau phases of infusion test. Wilcoxon test was applied to assess whether there was any significant difference between baseline and plateau phases for each estimator and calculated variables.

## Results

Table 1 presents all estimated physiological variables used for nICP estimations, at baseline and plateau phases, and their ∆ correlations with ∆ICP. Table 2 presents comparisons among non-invasive methods adopted in this study.

**Table 1:** Median values (IQR), 95 % CI for prediction of ICP and bias (in mmHg) are described for baseline and plateau phases. Spearman correlation between ∆ICP vs ∆nICP and averaged correlation across time during ICP increase (*n* = 53). At the 0.05 level, baseline and plateau distributions of nICPs and ICP are significantly different

Method	Baseline			Plateau			*R*	*R*
	Median (IQR)	95 % CI	Bias	Median (IQR)	95 % CI	Bias	(∆ICP vs ∆nICP)	(time domain)
*nICP_BB*	10.76 (15.08-7.30)^a^	15.33	4.46^b^	14.86 (20.1-11.26)^a^	19.21	−7.35^b^	0.30^c^	0.39 ± 0.43
*nICP_FVd*	16.97 (22.56-11.64)^a^	25.19	11.90^b^	21.74 (32.85-14.15)	29.97	1.66	−0.17	0.35 ± 0.41
*nICP_CrCP*	18.34 (20.38-14.89)^a^	15.09	11.12^b^	19.65 (23.80-16.92)	17.80	−2.53^b^	0.21	0.29 ± 0.24
*nICP_PI*	16.57 (17.46-16.06)^a^	10.58	8.91^b^	17.12 (17.73-16.40)^a^	19.07	−6.18^b^	0.45^c^	0.39 ± 0.40
*ICP*	7.74 (11.06-2.95)	-	-	22.13 (29.77-16.41)	-	-	-	-

^a^ At the 0.05 level, distributions between nICP and ICP are significantly different

^b^ The population mean is significantly different with the test mean (zero)

^c^ *Spearman* correlation coefficient is significant at the 0.05 level

**Table 2:** Median (IQR) values for all physiological variables estimated during baseline and plateau phases, with their respective ∆correlations with ∆ICP (*R*)

Variable	Baseline	Plateau	*p* value	*R* (∆ICP)	*R* (∆ABP)
ICP	7.77 (11.06-2.95)	22.13 (29.77-16.41)^a^	2.2 × 10^−16^	-	0.1
ABP	89.68 (101.5-78.23)	96.08 (121–87.07)^a^	5.6 × 10^−6^	0.11	-
HR	67.86 (77.96-61.98)	70.12 (79–60.83)	0.39	0.15	0.28^b^
CPP	82 (98.67-73.27)	79.40 (96.53-67.14)^a^	7.9 × 10^−4^	−0.38^b^	0.82^b^
FV	54.2 (66.27-42.47)	49.76 (61.92-39.07)^a^	8.4 × 10^−6^	0.14	−0.20
FV_s_	81.61 (101.6-66.76)	79.97(98.65-63.35)	0.07	−0.03	−0.21
FV_d_	33.17 (43.86-27.32)	31.93 (40.61-23.57)^a^	1.4 × 10^−6^	−0.22	0.21
PI	0.84 (1.05-0.76)	0.99 (1.13-0.83)^a^	1.1 × 10^−9^	0.45^b^	−0.04
CVR	1.64 (1.95-1.22)	1.55 (2.05-1.13)	0.56	−0.21	0.62^b^

^a^ At the 0.05 level, distributions between baseline and plateau are significantly different

^b^ *Spearman* correlation coefficient is significant at the 0.05 level

Median values of ICP and nICP are presented with their respective interquartile ranges (IQRs) in mmHg. Mann–Whitney test revealed significantly different distributions between all nICP estimators and ICP for baseline; for plateau phase only *nICP_BB* and *nICP_PI* presented such differences. Wilcoxon test revealed significantly different distributions between baseline and plateau nICPs and ICP.

Regarding confidence intervals (Bias ± 95 % CI), *nICP_BB* showed 15.33 ± 4.46 mmHg; *nICP_FVd* showed 25.19 ± 11.90 mmHg; *nICP_CrCP* showed 15.09 ± 11.12 mmHg and *nICP_PI* showed 10.58 ± 8.91 mmHg. During plateau every method presented increased CI: 19.21 ± −7.35 mmHg for *nICP_BB*; 29.97 ± 1.66 mmHg for *nICP_FVd*; 17.80 ± −2.53 mmHg for *nICP_CrCP* and 19.07 ± −6.18 mmHg for *nICP_PI*.

Correlations between ∆ICP and ∆nICP were better represented by *nICP_PI* and *nICP_BB*, with 0.45 (*p* = 0.0007) and *R* = 0.30 (*p* = 0.03), respectively. The other methods presented inferior and non-significant correlations: −0.17 (*p* = 0.21), 0.21 (*p* = 0.13) for *ICPn_FVd*, *nICP_CrCP*, respectively.

**Fig. 1:**
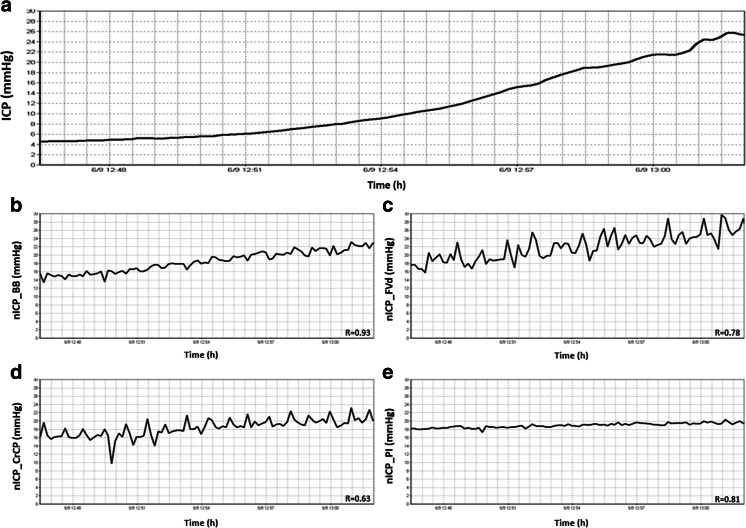
Example of good recording of nICP with four investigated methods when ICP changed considerably during the infusion test. **a** ICP; **b** *nICP_BB*; **c** *nICP_FVd*; **d** *nICP_CrCP*; **e** *nICP_PI*. *R* represents correlation coefficient between ICP and nICP (*R* = 0.93 for *nICP_BB*; *R* = 0.78 for *nICP_FVd*; *R* = 0.63 for *nICP_CrCP*; *R* = 0.81 for *nICP_PI*)

**Fig. 2:**
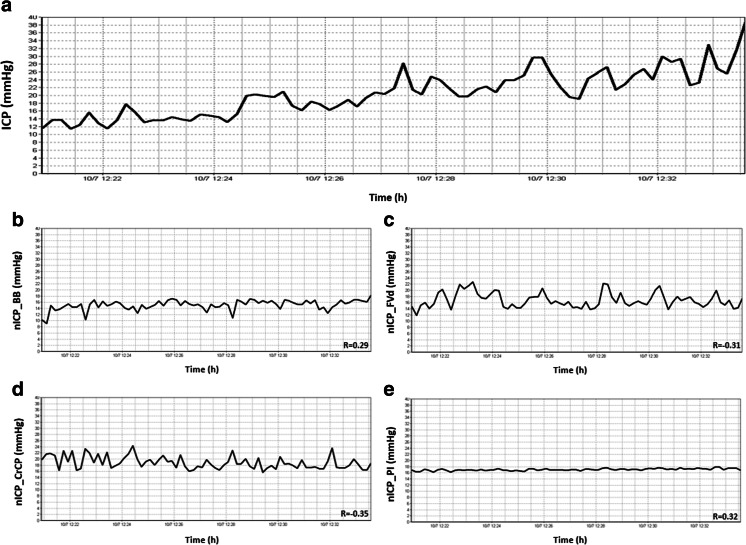
Example of poor recording of nICP with four investigated methods when ICP changed considerably during the infusion test. **a** ICP; **b** *nICP_BB*; **c** *nICP_FVd*; **d** *nICP_CrCP*; **e** *nICP_PI*. *R* represents correlation coefficient between ICP and nICP (*R* = 0.29 for *nICP_BB*; *R* = −0.31 for *nICP_FVd*; *R* = −0.35 for *nICP_CrCP*; *R* = 0.32 for *nICP_PI*)

Regarding variations of nICP in time domain during ICP increase *nICP_PI*, *nICP_BB* and *nICP_FVd* presented similar averaged correlations, 0.39 ± 0.40, 0.39 ± 0.43 and 0.35 ± 0.41, respectively. However, *nICP_CrCP* presented a smaller correlation (*R* = 0.29 ± 0.24). To demonstrate such variations and correlation between nICP and ICP in time, examples of a good and a poor recording of nICP with the four investigated methods when ICP changed considerably during the infusion test are showed in Figs. 1 and 2, respectively.

## Discussion

TCD has been used to estimate nICP in several conditions; however, their relative accuracy varies between different types of intracranial hypertension: vasogenic, CSF circulatory or secondary to brain volumetric changes (oedema, contusion, haematoma, etc.). Schmidt et al. [14] reported a CI for prediction of 12.8 mmHg, and Cardim et al. [3] a CI of 9.94 mmHg for *nICP_BB*. Kashif et al. [9], also using a model based on ABP and FV, reported 15 mmHg (standard deviation of error (SDE) of 7.6 mmHg). Using TCD-derived PI, Bellner et al. [1] and Cardim et al. [3] reported a 95 % CI for prediction of 4.2 mmHg and 9.62 mmHg, respectively. Non-invasive ICP methods based on the concepts of FVd and CrCP were also assessed and presented with CI of 14.62 mmHg and 9.19 mmHg, respectively [3]. For the moment, these confidence intervals have mainly been described for traumatic brain injury patients, with the exception of PI, which reflected a cohort of patients with several intracranial disorders [1].

The assessment of TCD-based nICP estimators during infusion tests revealed a limited accuracy of assessment ICP non-invasively and discrepancy in estimation of ICP. Of the four studied estimators, relative changes in mean ICP were better associated with estimated ICP using only two methods. Best scenario was obtained with the method based on TCD pulsatility index, followed by the mathematical black-box model, whereas the other methods did not present satisfactory correlation with measured ICP. Considering trends of measured ICP in time, all nICP methods presented better performance and overall correlations were more balanced among them.

Considering absolute values of ICP, analysis showed significantly different distributions between all nICP methods and ICP for baseline, whereas during the plateau phase *nICP_FVd* and *nICP_CrCP* had the same distributions as ICP. This indicates that, with exception of these cases, the nICP estimators were not able to accurately predict the absolute measure of ICP during these phases of the infusion test.

Nevertheless, all paired comparisons between estimators for baseline and plateau showed significant differences, demonstrating that infusion test did produce a significant increase in nICP for every estimator. Although significant within the same method, these differences were disproportional when comparing measured ICP and nICP estimations. Such disproportion was reflected in the confidence intervals for prediction found for baseline and plateau phases. In regards to bias, a non-significant difference between non-invasive and invasive methods is desirable, which means that both methods are not different in rendering mean ICP values. In this case, with exception of *nICP_FVd* during plateau phase, all estimators presented significant biases.

Regarding correlations between ∆ICP and ∆nICP, such results demonstrate the ability of the nICP methods to detect the magnitude of changes in measured ICP. Even though considered moderate correlations, n*ICP_PI* and *nICP_BB* were the only estimators to present significant levels. For detection of trends in time, which does not consider ICP as a numeric value but the behaviour of the methods during an ICP increase, the methods also displayed moderate averaged correlations. They showed better agreement for *nICP_PI*, *nICP_BB* and *nICP_FVd*, and a weaker correlation for *nICP_CrCP*. Figures 1 and 2, respectively, represent good and poor examples of correlations in time among ICP and nICP estimators.

As observed, nICP estimation differed mostly in terms of prediction of absolute values of ICP during infusion tests and to a lesser extent in terms of detection of dynamic changes. Different nICP accuracies may be explained by each method’s specific characteristics. The *nICP_BB* method reflects ABP waveform being constantly modified by TCD characteristics, and then is mostly susceptible to changes of vasogenic origin (such as CVR) and consequently cerebral blood flow. The *nICP_FVd* method (Eq. 1) is mostly modulated by the factor FVd/FVm and ABP. The *nICP_CrCP* method (Eq. 2) is modulated by changes in CVR and also by ABP. For *nICP_PI*, any changes in the components of flow velocity (FVm, FVs and FVd) are reflected in the PI calculation and consequently in the nICP estimation.

The nature of ICP elevation during infusion test is attributed to an increase in CSF circulation due to a direct addition of volume into the CSF space. TCD ultrasonography, however, is a technique mainly capable of detecting cerebrovascular changes in the arterial bed [15] (in our case, specifically the MCA). Thus, theoretically, it would be expected that changes in ICP estimated with TCD-based methods would present better accuracy if they were of vasogenic origin, rather than caused by variations of pressure in the CSF compartment.

**Fig. 3:**
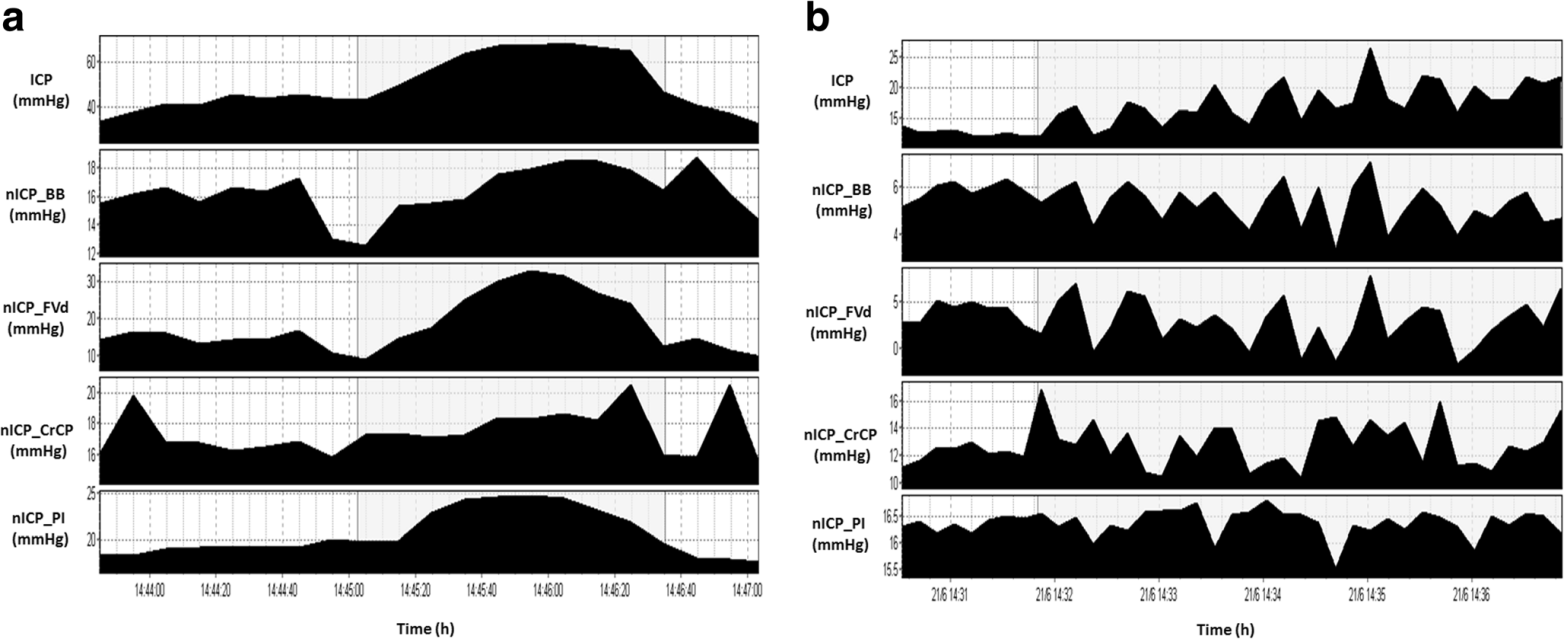
Example of vasogenic waves during infusion test. **a** *Shadowed area* represents a plateau wave of ICP. **b** *Shadowed area* represents B waves of ICP. It is possible to observe that at least for trends in time, there were good concordance between ICP and nICP methods

This can be exemplified by the fact that in specific cases where changes of ICP related to vasogenic fluctuations (plateau waves, B waves) overlapped the rise related to CSF infusion, the time-trend correlation between real and estimated ICP seemed to be remarkably better (as seen in Fig. 3), even with reliable replications of vasogenic waves patterns.

The way fluctuations in CSF circulation are transmitted to cerebral arterial bed might help understand the low accuracies presented by TCD methods during infusion test. In these circumstances, the rise in ICP originated from increased CSF circulation has direct and indirect influences on cerebral haemodynamics. The direct influence is represented by changes in CPP, altered directly by ICP. On the other hand, ICP has an indirect effect on cerebral haemodynamics, via changes in systemic haemodynamics. The changes in ABP observed during infusion tests are associated with an early Cushing response [13], in which rising ICP yields an increase in ABP. Therefore, transmission of fluctuations in CSF circulation to the cerebral arterial bed are primarily given by a decrease in CPP and secondarily by an increase in ABP.

In regard to haemodynamic and TCD-derived cerebrovascular variables used for the method’s estimations (ABP, HR, FV, FVs, FVd, PI and CVR), HR, FVs and CVR did not differ between plateau and baseline phases. Within the variables which presented significant difference, only changes in CPP and PI (∆CPP and ∆PI) were significantly correlated with changes in ICP. This finding is in agreement with results obtained by Bellner et al. [1], in which PI was strongly correlated with changes in ICP. In our case, however, PI presented only a moderate correlation with ICP, and was mainly modulated by FVd, as FVs did not present significant changes between baseline and plateau.

In this study, changes in ABP were significantly correlated to changes in CVR. This signifies that although such changes were not significant between baseline and plateau phases, this secondary increase in ABP produced by an early Cushing response caused minor vasogenic changes in cerebral haemodynamics [13]. This could have contributed to the better accuracy in cases which patients presented changes in ICP of vasogenic origin (Fig. 3).

Consequently, the low accuracy for prediction of ICP observed for TCD-based methods might be related to the nature of ICP elevation during infusion tests. Although increased CSF circulation is able to produce secondary changes on cerebral haemodynamics, this might not be the ideal manner to produce global changes of vasogenic origin that could be detected more accurately by TCD. Therefore, under these conditions, TCD cannot be considered a suitable technique for nICP estimation in conditions of increased CSF circulation.

In conclusion, changes of ICP observed during the test were expressed by nICP values with only moderate correlations. Vasogenic components of ICP seemed to be easier to estimate with TCD, than component related to increased CSF circulation. In this context, out of the four methods assessed, *nICP_PI* was the one with best performance for predicting changes in ∆ICP during infusion test, followed by *nICP_BB*. Methods based on FVd and CrCP showed unreliable correlations.

### Limitations

An intrinsic limitation of this study is that it considers analysis of retrospective data, collected at two different time points (1994–1998 and 2006). The majority of data was collected in the first period (*n* = 39, 73.6 %); nevertheless, the methodology and equipment used did not differ between the collection points. However analysis of retrospective data may evidence downsides of the study design, such material is unique and contains important information about the cerebrovascular dynamics of normal pressure hydrocephalus (NPH) patients during CSF infusion tests, which may be useful in different scenarios and applications.

A well-known limitation in studies approaching cerebral blood flow velocity measured with TCD is the inter- and intra-operator variability, as demonstrated by McMahon et al. [12]. Here, we attempted to minimise it by allowing only two experienced operators (M.C. and Z.C.) to collect the data. It is then assumed for this study that such variability was minimal.

A potential limiting factor to this study could be the non-invasive measurement of ABP. Although the response bandwidth obtained with Finapres® is adequate to register the ABP waveform with reliable accuracy, the supporting applications in which waveform-dependent indices are extracted [4] (such as in *nICP_BB* method), relative accuracy in terms of mean values of ABP did not show to be very precise. In a previous study, Stokes et al. [16] performed a comparison of invasive and non-invasive ABP measurements (specifically using Ohmeda Finapres® 2300), and found an inter-individual variability in absolute pressure readings. In our case, this could promote a misestimation for *nICP_FVd* and *nICP_CrCP*, which present ABP as multiplier in the formula.

Another limiting factor could be the origin of the methods used for nICP estimations, as all of them were derived from cohorts of traumatic brain injury patients. Different physiological mechanisms leading to ICP fluctuations in TBI and hydrocephalus might play a significant role in the way ICP was estimated.
